# 2718. Prevalence of Infections Following Anti-CD30 Targeted Chimeric Antigen Receptor T-cell Therapy for Relapsed and Refractory Lymphoma

**DOI:** 10.1093/ofid/ofad500.2329

**Published:** 2023-11-27

**Authors:** Michael C Mohnasky, Jonathan Huggins, Julia A Messina, Manish K Saha, Jennifer Saullo, Melody Smith, Joseph Stromberg, Megan Walsh, Tessa Andermann

**Affiliations:** University of North Carolina at Chapel Hill, School of Medicine, Chapel Hill, North Carolina; Duke University Hospital, Durham, North Carolina; Duke University, Apex, North Carolina; University of North Carolina, Chapel Hill, Chapel Hill, North Carolina; Duke University, Apex, North Carolina; Stanford University, Stanford, California; University of North Carolina, Durham, North Carolina; UNC School of Medicine, Wilmington, North Carolina; University of North Carolina, Durham, North Carolina

## Abstract

**Background:**

Chimeric antigen receptor (CAR) T-cell therapy has revolutionized the treatment of relapsed or refractory hematologic malignancies. Clinical trials have demonstrated anti-CD30 CAR T-cell efficacy in patients with CD30+ malignancies such as Hodgkin lymphoma, but little is known about post-treatment infections. Understanding infections in this context is necessary to inform antimicrobial prophylaxis and treatment guidelines. The purpose of this project was to provide the first known characterization of infections in patients who received anti-CD30 CAR T-cell therapy.

**Methods:**

Ours was a retrospective cohort study of adult patients receiving anti-CD30 CAR-T cells between 2016 and 2021. For the year following therapy, we tracked viral, bacterial, and fungal infections with both a microbiological confirmation or strong clinical suspicion of infection.

**Results:**

62 patients received anti-CD30 CAR T-cell therapy during the study period. The median age was 40.9 years, 35.5% were female, 79.0% were of European ancestry, and 93.5% were Not Hispanic or Latino. 77.4% of patients’ primary malignancy was Hodgkin’s lymphoma. 56.5% received Fludarabine and Bendamustine as the preparatory regimen (Table I). Five patients were lost to follow-up and were not included in infection analysis. Overall, there were 37 infectious events in 25 patients (25/57, 43.9%). 43.2% of infections happened within 30 days, 35.1% within 31-90 days, and 21.6% within 90 days to one year. Viral infections were the most common overall (48.6%) and within each time period (Table II). The most common pathogen was Varicella Zoster (10.8%); none of these patients were on valacyclovir prophylaxis at the time of infection. The lung was the most common site of infection overall (29.7%) (Figure 1). Death due to infection was seen in one patient during the study period.

**Figure:**
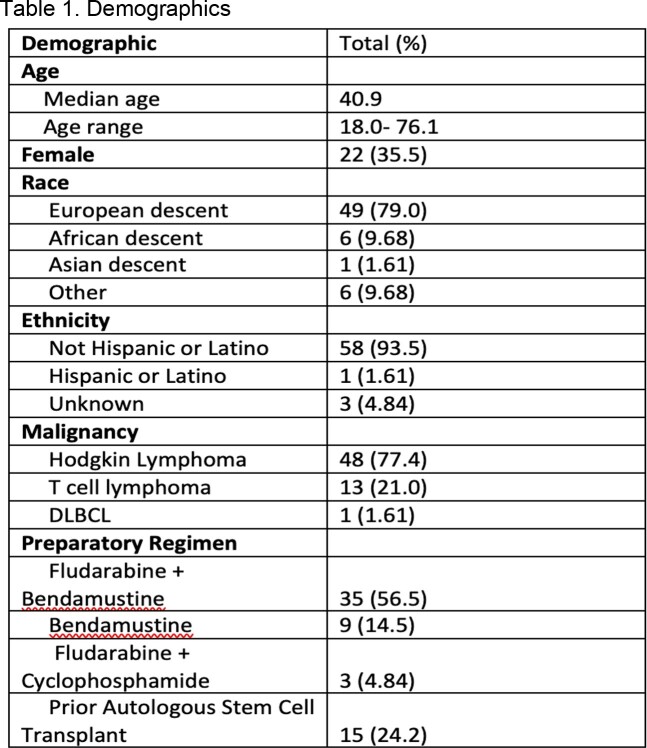

Table showing the demographic characteristics of the study cohort including age, gender, race, ethnicity, malignancy, and preparatory regimen.

**Figure:**
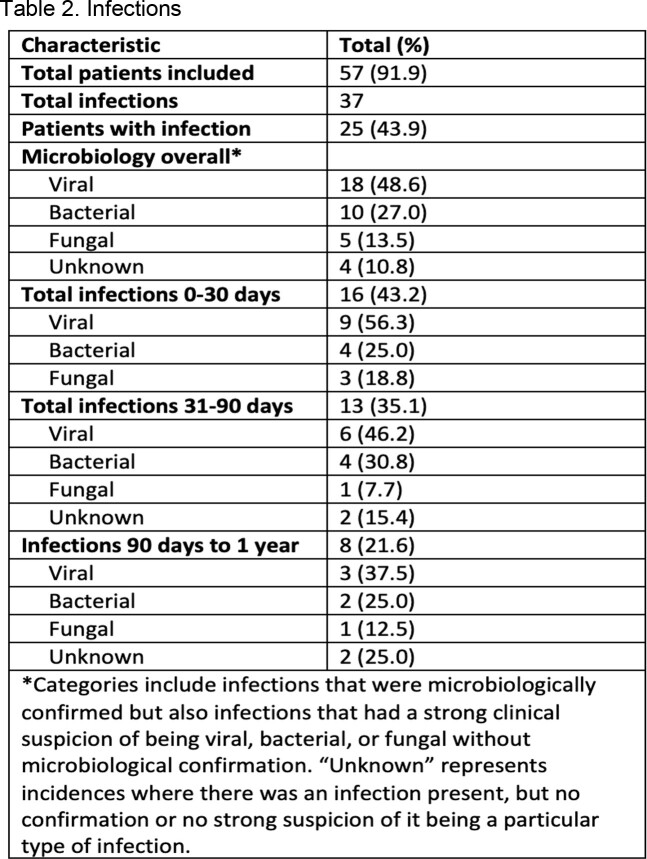

Table capturing infection characteristics including total infections, patients with an infection, and microbiology of the infections overall and broken down by time period.

**Figure 1.**
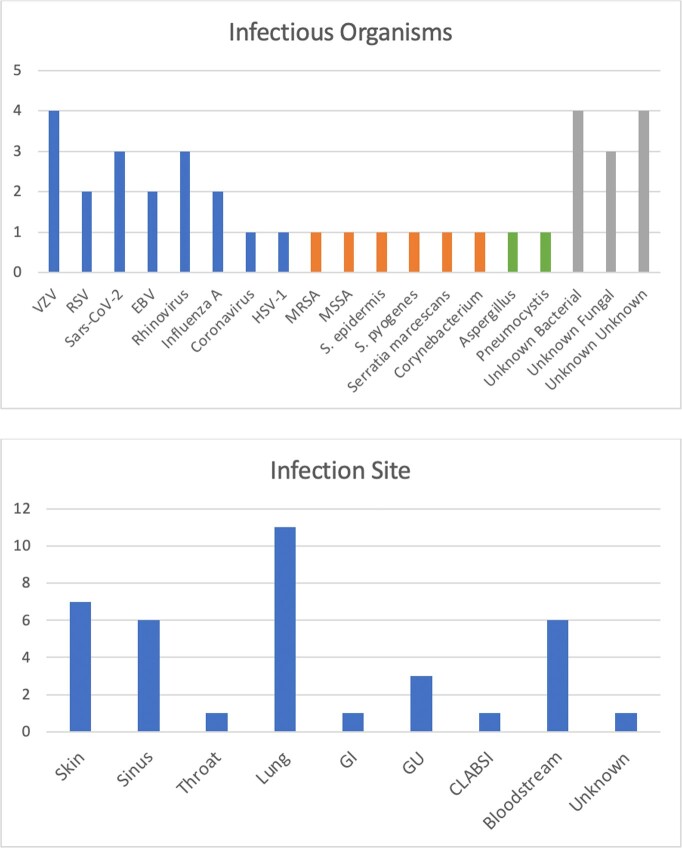
Infectious Organisms and Site of Infection

Two bar charts with the top bar chart demonstrating the frequency of each infectious organism and the bottom bar chart demonstrating the frequency of each infection site.

**Conclusion:**

Infections following anti-CD30 CAR T-cell therapy are most common within 30 days following therapy though risk persists through one-year post-therapy. Viral infections were the most prevalent which differs from reports with anti-CD19 CAR T-cell therapy in which bacterial infections were most common. These results may inform future antimicrobial guidelines for patients receiving these therapies.

**Disclosures:**

**Manish K. Saha, MD**, Novavax: Stocks/Bonds **Melody Smith, MD, MS**, BMS: Advisor/Consultant

